# 伴t(17;19)(q21-22;p13)/TCF3-HLF急性淋巴细胞白血病7例临床分析

**DOI:** 10.3760/cma.j.cn121090-20240220-00069

**Published:** 2024-09

**Authors:** 妍 浦, 吟 刘, 湘粤 周, 宝全 宋, 剑 张, 婉惠 颜, 谦 王, 建农 岑, 宏杰 沈, 琴荣 王, 苏宁 陈, 金兰 潘, 惠英 仇

**Affiliations:** 苏州大学附属第一医院血液内科，江苏省血液研究所，国家血液系统疾病临床医学研究中心，苏州 215006 The First Affiliated Hospital of Soochow University, Jiangsu Institute of Hematology, National Clinical Research Center for Hematologic Diseases, Suzhou 215006, China

## Abstract

回顾性分析2017年6月至2022年8月苏州大学附属第一医院收治的7例TCF3-HLF融合基因阳性的急性B淋巴细胞白血病（B-ALL）患者的临床资料，总结其临床特征及预后。7例B-ALL患者中男4例，女3例，中位年龄18（11～33）岁。5例患者CD33表达阳性，4例患者为正常染色体核型。2例患者初诊时合并高钙血症，1例患者复发时出现高钙血症，6例患者初诊时合并凝血功能障碍。7例患者接受诱导化疗后5例达完全缓解，其中4例后续复发。2例患者经2次诱导化疗后仍未缓解，其中1例接受贝林妥欧单抗免疫治疗后达完全缓解。3例患者接受嵌合抗原受体T细胞治疗，3例患者后续接受造血干细胞移植。5例患者死亡，2例患者存活且持续完全缓解。TCF3-HLF阳性B-ALL罕见，复发率高，预后差。

## Abstract (en)

A retrospective analysis of the clinical data of seven acute B-lymphoblastic leukemia（B-ALL）patients with TCF3-HLF fusion gene-positive admitted to the First Affiliated Hospital of Soochow University from June 2017 to August 2022 was conducted to summarize their clinical features and prognoses. The seven B-ALL patients comprised four males and three females, with a median age of 18（11–33）years. Five patients tested positive for CD33 expression, and four patients had a normal karyotype. Two patients had hypercalcemia at the initial diagnosis, and one patient developed hypercalcemia at relapse. Six patients presented with coagulation dysfunction at diagnosis. After induction chemotherapy, five out of seven patients achieved complete remission, of which four subsequently relapsed. Two patients did not achieve remission even after two rounds of induction chemotherapy, with one achieving complete remission after treatment with blinatumomab immunotherapy. Three patients underwent chimeric antigen receptor T cell therapy, whereas three patients subsequently underwent hematopoietic stem cell transplantation. Five patients died, while two patients survived with sustained complete remission. TCF3-HLF-positive B-ALL is rare and has a high relapse rate and poor prognosis.

在急性B淋巴细胞白血病（B-ALL）中，细胞及分子遗传学异常与亚型、复发风险和预后密切相关。TCF3-HLF是B-ALL中罕见的融合基因，具有高度侵袭性，世界卫生组织（WHO）第5版血液淋巴细胞肿瘤分型将B-ALL伴TCF3-HLF融合列为一个独立亚型[1]。TCF3-HLF由t（17;19）（q21-22;p13）易位形成，即19p13断裂位点的TCF3基因与17q21断裂位点的HLF基因融合。B-ALL伴TCF3-HLF融合基因常合并高钙血症和凝血功能障碍，即使接受造血干细胞移植治疗仍预后不良[2]–[3]。由于伴TCF3-HLF的B-ALL罕见且预后较差，其最佳治疗方案尚无定论。本研究回顾性分析了苏州大学附属第一医院收治的7例伴t（17;19）（q21-22;p13）/TCF3-HLF的B-ALL患者的临床特征、治疗及疗效。

## 病例与方法

1. 病例：本研究纳入苏州大学第一附属医院2017年6月至2022年8月收治的7例伴TCF3-HLF的B-ALL患者。诊断根据2022版WHO造血和淋巴组织肿瘤的分型诊断标准。患者经骨髓细胞形态学、白血病免疫分型、细胞遗传学、分子生物学等检查确诊。

2. 细胞遗传学分析：从未接受刺激的24 h骨髓细胞培养物中制备R显带中期细胞，使用标准方法进行常规染色体分析。根据国际人类细胞基因组命名系统（ISCN，2020）描述克隆核型异常。每个标本至少观察到10个中期分裂象。使用一组单克隆抗体（法国Immunotechg公司产品）通过流式细胞仪（美国Beckman Coulter公司产品）进行免疫表型分析，B细胞标志物为CD10、CD19和CD20，T细胞标志物为CD2、CD3、CD5和CD7；骨髓标志物为CD13、CD14、CD15和CD33，造血干细胞标志物为CD34。

3. 分子生物学分析：通过转录组测序（RNA-Seq）鉴定B-ALL中的融合基因。使用标准方案提取总RNA，并用KAPA RNA HyperPrep试剂盒（德国Roche公司产品）评估完整性。应用Illumina NextSeq 550系统（美国Illumina公司产品）对产生的文库进行测序。使用STAR-fusion软件（1.9.0版）对融合基因进行分析。融合基因通过参数（跨越多个外显子的完整序列数目、包含融合连接位点的完整序列数目等）过滤。

4. 治疗方案及疗效评估：7例患者均接受多药联合诱导化疗，5例患者诱导化疗后达完全缓解（CR），继续接受多药联合巩固化疗，4例患者后续复发。6例患者（包括4例复发及2例诱导治疗后未缓解患者）接受多药联合再诱导化疗，其中3例患者再诱导化疗后接受嵌合抗原受体T（CAR-T）细胞治疗，1例患者再诱导化疗后接受贝林妥欧单抗免疫治疗。3例患者后续接受异基因造血干细胞移植（allo-HSCT）治疗。疗效评估参照《中国成人急性淋巴细胞白血病诊断与治疗指南（2021年版）》[4]。

5. 随访：采用电话、查阅患者住院病历及门诊病历的方式随访，随访截止日期为2024年1月1日，中位随访时间为13（5～73）个月，所有患者均未失访。

6. 统计学处理：采用R（4.2.3）软件进行统计学分析。计量资料用中位数（范围）表示，计数资料用例数（百分比）表示。采用Kaplan-Meier法描述患者的生存情况。

## 结果

1. 临床特征：7例伴TCF3-HLF的B-ALL患者中男4例，女3例，中位年龄19（11～33）岁（表1）。初诊时血常规：WBC 15.6（7.5～88.7）×10^9^/L，HGB 103（44～159）g/L，PLT 51（7～270）×10^9^/L。初诊时骨髓原始细胞比例82.5％（41％～92％）。2例患者（例3、4）初诊时合并高钙血症，1例患者（例7）复发时出现高钙血症。6例患者（例1、3～7）初诊时合并凝血功能障碍。7例患者免疫表型CD10、CD19阳性，其中6例患者表达cCD79a，5例患者表达CD33，4例患者（例1～4）表达CD38、DR。1例患者（例4）免疫表型的分化阶段为普通型B-ALL，其余6例患者均为前体B-ALL。4例患者为正常染色体核型，例5为add（19）（p13），例7为9p−、9q+、12q−和der（14），仅例6出现t（17;19）（q21;p13）染色体异常（图1）。7例患者均通过逆转录聚合酶链反应（RT-PCR）检出TCF3-HLF融合基因，2例患者（例6、7）进一步行转录组测序（RNA-Seq），TCF3-HLF是TCF3基因第15外显子与HLF基因第4外显子融合。7例患者二代测序共检出7种、11个基因突变（表1）。

**表1:** 7例伴TCF3-HLF的急性B淋巴细胞白血病患者的临床特征

例号	年龄(岁)	性别	临床表现	WBC（×10^9^/L）	骨髓原始细胞比例	免疫表型	分化阶段	染色体核型	RT-PCR	RNA-seq	基因突变
1	17	男	皮肤瘀点、瘀斑、肛周疼痛	15.6	81.9%	CD10、CD19、CD13、CD33、CD38、cCD79a	Pre B-ALL	正常	TCF3::HLF	否	SETD2、KRAS
2	18	男	腰部疼痛	7.5	41.0%	CD33、CD10、CD19、CD38、cCD79a、CyTDT	Pre B-ALL	正常	TCF3::HLF	否	SETD2、KRAS、NOTCH1
3	30	男	腰部疼痛、骨痛、下肢活动受限	88.7	85.0%	DR、CD10、CD19、CD38、cCD79a	Pre B-ALL	正常	TCF3::HLF	否	阴性
4	11	男	乏力	11.4	92.0%	CD45、CD10、CD34、CD19	Common B-ALL	正常	TCF3::HLF	否	KRAS
5	33	女	咳嗽、咳痰、皮肤瘀点、瘀斑	61.6	42.5%	CD10、CD19、DR、cCD79a、CD33、CD22、MPO	Pre B-ALL	46,XX,add(19) (p13)	TCF3::HLF	否	KRAS、CDKN2A、BCOR
6	26	女	发热、乏力、皮肤瘀点、瘀斑	53.0	82.5%	CD38、CD33、CD123、CD22、CD19、CD10、CD9、CyTDT、cCD79a、DR	Pre B-ALL	46,XX,t(17;19) (q21;p13)	TCF3::HLF	e4和e15	CRLF2
7	19	女	口周麻木、脾脏肿大	7.7	92.0%	DR、CD10、CD19、CD33、cCD79a	Pre B-ALL	46,XX,9p−,9q+,12q−,der(14)	TCF3::HLF	e4和e15	SMC2

**注** RT-PCR：逆转录聚合酶链反应；RNA-seq：转录组测序；Pre B-ALL：前体急性B淋巴细胞白血病；Common B-ALL：普通型急性B淋巴细胞白血病

**图1:**
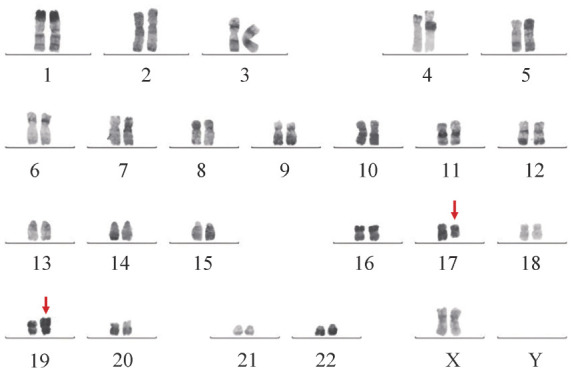
例6染色体核型结果

2. 治疗及疗效：7例患者均接受多药联合诱导化疗，1个疗程诱导化疗后形态学CR率为71.4％（5/7），微小残留病阴性率为28.6％（2/7），7例患者均未发生肿瘤溶解综合征。2例患者（例5、6）接受2次诱导化疗后仍未缓解，其中例6接受贝林妥欧单抗免疫治疗后达CR，后续接受了allo-HSCT，但最终因复发死亡。5例在初次诱导化疗后达CR的患者继续接受多药联合巩固化疗，其中例2持续CR状态并接受allo-HSCT，4例患者（例1、3、4、7）复发并接受多药联合再诱导化疗，仅例1在复发后经过治疗第2次达CR状态（CR_2_）。3例患者（例1、4、7）后续接受了CAR-T细胞治疗，均达CR，但CR持续时间较短，分别在CAR-T细胞治疗后22 d、70 d、74 d再次复发，例1接受挽救性造血干细胞移植。例1、例7出现3级细胞因子释放综合征，例4出现1级细胞因子释放综合征。中位随访时间13（5～73）个月，2例患者（例1、2）存活，5例患者（例3～7）死亡。7例患者的治疗经过及生存情况见表2。

**表2:** 7例伴TCF3-HLF的急性B淋巴细胞白血病患者的治疗、疗效及生存情况

例号	诱导化疗方案/疗效	巩固化疗方案/疗效	再诱导化疗方案/疗效	CAR-T细胞治疗/疗效	移植	1个疗程诱导治疗达CR	1个疗程诱导治疗MRD阴性	生存情况（死亡原因）	OS期（月）
1	CIVP/CR	CAM/CR；Hyper-CVAD-B+L-asp/复发	MTX、VDS、Mito、Vp16、Dex/CR_2_；IVP+Ven/CR_3_	是/CR_2_，22 d后复发	是	是	否	生存	69.0
2	CIVP/CR	Hyper-CVAD-B/CR；Hyper-CVAD-A+L-asp/CR	否	否	是	是	否	生存	73.0
3	VDLP/CR	CAM+L-asp/CR；VDLP/复发	HAG/NR；AZA+Ven/NR	否	否	是	是	死亡（感染性休克）	9.0
4	VDLP/CR	CAM、MTX/CR；CIVP/CR；CAM/复发	VDCLP/NR	是/CR_2_，70 d后复发	否	是	否	死亡（复发）	13.0
5	Hyper-CVAD-A/NR	否	FLAG/NR	否	否	否	否	死亡（复发）	5.0
6	C-IDOP/NR	否	MVP/NR	否	是	否	否	死亡（复发）	5.0
7	IVP+L-asp/CR	CAML/CR；HD-MTX、VDS/CR；CTX、IVP/CR；CTX、Vp16/复发	MD Ara-C/NR	是/CR_2_，74 d后复发	否	是	是	死亡（复发）	12.9

**注** CIVP：环磷酰胺+伊达比星+长春地辛+地塞米松；VDLP：长春新碱+柔红霉素+门冬酰胺酶+地塞米松；Hyper-CVAD-A：环磷酰胺+长春新碱+阿霉素+地塞米松；C-IDOP：环磷酰胺+伊达比星+柔红霉素+长春新碱+地塞米松；IVP：伊达比星+长春地辛+泼尼松；L-asp：左旋门冬酰胺酶；CAM：环磷酰胺+阿糖胞苷+6-巯基嘌呤；Hyper-CVAD-B：甲氨蝶呤+阿糖胞苷；MTX：甲氨蝶呤；CAML：环磷酰胺+阿糖胞苷+培门冬酶+6-巯基嘌呤；HD-MTX：大剂量甲氨蝶呤；VDS：长春地辛；CTX：环磷酰胺；Vp16：依托泊苷；Mito：米托蒽醌；Dex：地塞米松；Ven：维奈克拉；HAG：高三尖杉酯碱+阿糖胞苷+粒细胞集落刺激因子；AZA：阿扎胞苷；VDCLP：长春新碱+柔红霉素+环磷酰胺+门冬酰胺酶+泼尼松；FLAG：氟达拉滨+阿糖胞苷+粒细胞集落刺激因子；MVP：米托蒽醌+长春新碱+地塞米松；MD Ara-C：中剂量阿糖胞苷；CR：完全缓解；NR：未缓解；CAR-T细胞：嵌合抗原受体T细胞；MRD：微小残留病；OS：总生存

## 讨论

伴t（17;19）（q21;p13）/TCF3-HLF的急性淋巴细胞白血病（ALL）较为罕见，少数患者为成年人[5]。仅60％～70％的ALL患者核型分析可检出染色体数目和结构异常，原因包括细胞培养失败、染色体形态学不良导致可分析的中期分裂象数量有限、分子水平的插入或易位等[6]–[9]。本研究包括6例成年人和1例儿童，7例患者中仅1例（例6）的染色体核型为t（17;19）（q21;p13），4例患者为正常染色体核型，2例患者通过R显带或G显带可检测到17和19号染色体易位形成的两条衍生染色体（17q−和19p+）及der（17）、der（19）异常。4例染色体核型正常的患者中有2例（例1、2）持续CR至今，总生存时间分别为69、73个月，提示伴TCF3-HLF而染色体核型正常的ALL患者可能预后更好。所有患者均采用RT-PCR和（或）RNA-Seq检测出TCF3-HLF融合基因。因此，传统的显带技术可能无法检测TCF3-HLF融合基因的真实发生率，应结合荧光原位杂交、RT-PCR或RNA-seq等分子学检测技术以免漏诊。

TCF3-HLF阳性ALL常见的临床特征包括凝血功能障碍、高钙血症和骨髓标志物的异常表达[10]–[11]，凝血功能障碍在B-ALL中并不常见，且伴TCF3-HLF的B-ALL患者出现凝血功能障碍的机制尚未明确。有研究认为，ALL伴TCF3-HLF的高钙血症部分由甲状旁腺激素相关肽介导，高钙血症并不影响B-ALL的预后[6],[12]，本研究7例患者中6例在初诊时发现凝血功能障碍，2例在初诊时发现高钙血症，1例在复发时发现高钙血症，与文献报道中TCF3-HLF阳性ALL的临床特征相符。本研究中3例合并高钙血症的患者最终均死亡，提示TCF3-HLF阳性ALL合并高钙血症可能预后不佳。本研究7例B-ALL患者中5例CD33阳性，1例儿童患者（例4）免疫表型的分化阶段为普通型B-ALL，其余6例成人患者均为前体B-ALL，提示B-ALL中t（17;19）易位可能与CD33表达有关。

TCF3-HLF嵌合体由TCF3因子的转录激活域和HLF因子的DNA结合域组成。TCF3是驱动B淋巴细胞发育的转录因子，HLF是参与维持造血干细胞库所必需的因子[13]–[15]。研究显示PAR蛋白与预后不良有关，HLF基因编码的蛋白质与其他PAR家族成员形成同源二聚体或异源二聚体，并与序列特异性启动子位点结合，可能是TCF-HLF阳性ALL患者预后不良的机制之一[16]–[17]。

TCF3-HLF融合基因本身不足以发生肿瘤转化，基因突变等其他遗传因素也可能导致肿瘤发生[18]–[19]。SETD2突变在急性髓系白血病、骨髓增生异常综合征等疾病中提示预后不良及化疗耐药，而Contreras Yametti[20]等的研究表明SETD2突变在ALL中并非驱动克隆进化和复发的关键因素。Fischer等[3]的研究表明，尽管在TCF3-HLF阳性的B-ALL患者中观察到RAS信号通路基因（如KRAS、NRAS和PTPN11），但RAS突变可能不是疾病进展的必要条件。本研究通过二代测序技术检测7例患者的基因突变，检出包括KRAS、SETD2、NOTCH1、CDKN2A、BCOR、CRLF2和SMC2在内的基因突变，在4例患者（例1、2、4、5）中检测到KRAS突变，在2例（例1、2）患者中检测到SETD2突变。本研究中2例伴SETD2和KRAS突变的患者（例1、2）生存至今且持续CR状态。上述突变对TCF3-HLF阳性B-ALL发病机制及预后的影响有待进一步研究。

伴TCF3-HLF的B-ALL预后差，对常规化疗耐药，最佳治疗方案尚无定论[3]。多数患者在确诊后2年内复发或死亡，5年无复发生存率为0[11]。因此，应考虑采用新的免疫治疗改善患者预后。Wu等[21]报道了CAR-T细胞治疗对伴TCF3-HLF的儿童ALL的有效性。Ahmed等[22]报道，1例伴TCF3-HLF的B-ALL患者在CAR-T细胞治疗后获得CR，3个月后出现复发。本研究中有3例患者（例1、4、7）接受了CAR-T细胞治疗，例1、例4、例7分别在第12天、第31天、第36天达到CR，但CR持续时间短，3例患者分别在CAR-T细胞治疗后第22天、第70天、第74天复发，例4和例7最终死亡，例1接受挽救性移植后生存至今。本研究的结果支持CAR-T细胞对于减轻肿瘤负荷的有效性，但缓解持续时间短，均在早期出现复发，复发的主要原因可能是CAR-T细胞消失或CD19低表达[23]。Mouttet等[24]的研究表明，贝林妥欧单抗免疫治疗可以更好地清除残留病灶，使伴TCF3-HLF的B-ALL患者在造血干细胞移植（HSCT）前实现CR。本研究中例6接受两次诱导化疗均未缓解，接受贝林妥欧单抗免疫治疗后达到CR，后续桥接allo-HSCT。

TCF3-HLF可能在BCL2蛋白中有转录靶点，高水平抗凋亡癌蛋白BCL2引起的对细胞凋亡的抵抗可能会改善癌细胞的存活率，并代表一个可用药的靶点[25]–[26]。维奈克拉是一种高度选择性的BCL2基因抑制剂，BCL2基因通过阻止细胞程序性死亡增加细胞的存活率[26]。Glover等[27]的研究表明维奈克拉可能对TCF3-HLF阳性的ALL有效。然而，Lasica等[28]的研究表明，维奈克拉治疗TCF3-HLF阳性ALL后仍有复发可能，并不是治疗TCF3-HLF阳性ALL的金标准。本研究1例患者复发后接受维奈克拉联合化疗后达到CR，但最终复发，与文献报道相符[28]。Zhang等[2]报道，第1次CR后早期进行allo-HSCT可以延长伴TCF3-HLF的B-ALL患者的生存时间。本研究中3例患者（例1、2、6）接受了allo-HSCT治疗，2例患者（例1、2）移植后存活至今并持续缓解，1例患者（例6）在移植后复发。4例未接受allo-HSCT的患者及1例移植后复发的患者均死亡。本研究提示HSCT可能延长伴TCF3-HLF的B-ALL患者的无病生存期和总生存期。

总之，TCF3-HLF阳性B-ALL罕见，复发率高，预后差，免疫靶向治疗可能有助于清除残留肿瘤，但持续缓解时间短，尽早行HSCT可能有助于改善预后。本研究样本量较小，尚需进行更大规模的多中心研究进一步验证。
